# Role of Microglial M1/M2 Polarization in Relapse and Remission of Psychiatric Disorders and Diseases

**DOI:** 10.3390/ph7121028

**Published:** 2014-11-25

**Authors:** Yutaka Nakagawa, Kenji Chiba

**Affiliations:** 1 Research Strategy and Planning Department, Research Division, Mitsubishi Tanabe Pharma Corporation, Yokohama 227-0033, Japan; E-Mail: Nakagawa.Yutaka@mf.mt-pharma.co.jp; 2 Advanced Medical Research Laboratories, Research Division, Mitsubishi Tanabe Pharma Corporation, Yokohama 227-0033, Japan

**Keywords:** microglia, M1/M2 polarization, neuroinflammation, schizophrenia, major depressive disorder, dysfunction of neural network, endocannabinoids, lipid mediators

## Abstract

Psychiatric disorders such as schizophrenia and major depressive disorder were thought to be caused by neurotransmitter abnormalities. Patients with these disorders often experience relapse and remission; however the underlying molecular mechanisms of relapse and remission still remain unclear. Recent advanced immunological analyses have revealed that M1/M2 polarization of macrophages plays an important role in controlling the balance between promotion and suppression in inflammation. Microglial cells share certain characteristics with macrophages and contribute to immune-surveillance in the central nervous system (CNS). In this review, we summarize immunoregulatory functions of microglia and discuss a possible role of microglial M1/M2 polarization in relapse and remission of psychiatric disorders and diseases. M1 polarized microglia can produce pro-inflammatory cytokines, reactive oxygen species, and nitric oxide, suggesting that these molecules contribute to dysfunction of neural network in the CNS. Alternatively, M2 polarized microglia express cytokines and receptors that are implicated in inhibiting inflammation and restoring homeostasis. Based on these aspects, we propose a possibility that M1 and M2 microglia are related to relapse and remission, respectively in psychiatric disorders and diseases. Consequently, a target molecule skewing M2 polarization of microglia may provide beneficial therapies for these disorders and diseases in the CNS.

## 1. Introduction

Abnormalities of homeostasis lead to dysfunction in our body’s orchestration and subsequently induce development and relapse of disorders and diseases. The mechanisms of improvement, remission, and recovery of disorders and diseases are also based on orchestrated systems. Multiple sclerosis (MS) is the most common immune-mediated demyelinating disease in the central nervous system (CNS) [1,2]. There are approximately two million MS patients and two-third of the patients develop relapsing remitting MS, in which neurologic symptoms occur followed by partial or complete recovery [1,2]. Pathologically, the most obvious abnormalities of the CNS are characterized as demyelination of white matter associated with inflammatory cells, including T cells, B cells, and macrophages [3]. Many studies have revealed that T cells from MS patients preferentially target myelin antigens such as myelin basic protein, myelin oligodendrocyte glycoprotein, and myelin proteolipid protein [4]. Recent studies have indicated that myelin antigen-specific, interleukin (IL)-17-expressing CD4 T cells (Th17 cells) infiltrate into the CNS beyond blood brain barrier (BBB) and play a pathogenic role in MS [5,6]. Other studies have suggested that α4β1 integrin (VLA4), osteopontin, and αB crystallin play a key role in relapse and remission of MS [7]. An adhesion molecule, α4β1 integrin mediates T cell migration from the blood to CNS. Osteopontin binds to α4β1 integrin and stimulates pro-inflammatory cytokine production, whereas αB crystallin inhibits neuroinflammation in the CNS. Furthermore, it has been reported that imbalance of M1/M2 macrophages is involved in relapse of experimental autoimmune encephalomyelitis (EAE), a model for MS, and that M2 macrophages may contribute to amelioration of EAE [8].

It has been widely accepted that psychiatric disorders such as schizophrenia and major depressive disorder are caused by neurotransmitter abnormalities [9]. Patients with these disorders often experience relapse and remission as seen in MS patients. However, the underlying molecular mechanisms of relapse and remission still remain unclear. Recent advanced immunological analyses have revealed that M1/M2 polarization of macrophages plays an important role in controlling the balance between promotion and suppression in inflammation [8]. Microglial cells share certain characteristics with macrophages and contribute to immune-surveillance in the CNS [10,11]. Classically activated microglia (M1 polarized microglia) can produce pro-inflammatory cytokines, reactive oxygen species (ROS), and nitric oxide (NO), implying their contribution to neural network dysfunction in the CNS. On the other hand, alternatively activated microglia (M2 polarized microglia) can express cytokines and receptors that are implicated in inhibiting inflammation and restoring homeostasis. Several studies suggest that neuroinflammation is associated with psychiatric disorder and disease symptoms [12,13,14]; however, no effective explanation is proposed for the underlying molecular mechanisms of relapse and remission. In this review, we summarize current understanding of microglial immunoregulatory functions and provide a possibility that M1 and M2 microglia are related to relapse and remission, respectively, in psychiatric disorders and diseases.

## 2. M1/M2 Polarization of Macrophages and Microglia

Under tissue damage or infection conditions, macrophages originate from tissue-resident precursors or circulating monocytes that migrate to inflammation sites, whereas “patrolling” monocytes are later recruited to inflamed sites to resolve inflammatory process [15,16,17]. The diversity and plasticity of macrophages lead to the identification of several functional polarization states, which are ultimately dependent on the macrophage extracellular environment. Pathogen-associated molecular patterns (PAMPS) or damage-associated molecular patterns (DAMPS) can stimulate resting macrophages via toll-like receptors (TLRs) or ATP receptors, respectively [18]. Subsequently, the classical activation of resting macrophages leads to M1 macrophages in the presence of lipopolysaccharide (LPS) and type 1 helper T cell (Th1)-derived cytokine, interferon (IFN)-γ [18]. M1 macrophages can produce pro-inflammatory cytokines/mediators such as IL-1β, IL-6, tumor necrosis factor (TNF)-α, CC-chemokine ligand 2 (CCL2), ROS, and NO, and play a central role in host defense against bacterial and viral infections [19]. On the other hand, it has been demonstrated that type 2 helper T cell (Th2)-derived cytokines, IL-4 and IL-13, can induce alternative activation of macrophages to M2 (particularly ‘M2a’) phenotype [19]. M2a macrophages express arginase-1 (Arg-1), Ym1, CD36, CD163, and CD206 on the cell surface and produce anti-inflammatory cytokine, IL-10 which can suppress M1 macrophage-mediated inflammation [18,19]. Although there are known to be three different phenotypes of M2 macrophages (M2a, M2b, and M2c), these M2 phenotypes are thought to reflect a spectrum of plastic functional conditions rather than a set of discrete activation status [20].

M1 and M2 macrophages can be converted into each other in their specific microenvironment [21]. It has been noted that CCL2 and IL-6 are released in neurodegenerative and neuroinflammatory conditions and can induce M2 polarization of macrophages [22]. Many key transcription factors such as signal transducer and activator of transcription (STATs), interferon-regulatory factor (IRFs), nuclear factor (NF)-κB, activator protein 1 (AP1), peroxisome proliferator-activated receptor (PPAR)-γ, and c-AMP-responsive element-binding protein (CREB) are involved in macrophage polarization and these factors interact with each other, regulating macrophages to a certain phenotype in various inflammatory diseases [23]. Consequently, M1 and M2 macrophages represent two terminals of the full spectrum of macrophage activation. Transformation of different phenotypes of macrophages regulates the initiation, development, and cessation of inflammatory diseases.

**Figure 1:**
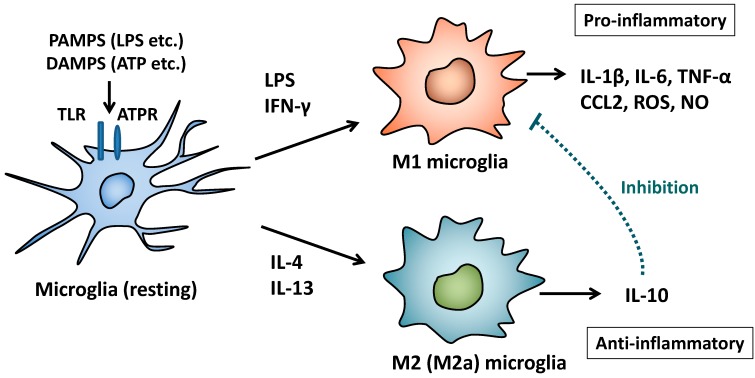
M1/M2 polarization of microglia and their immunoregulatory functions. Resting microglial cells are stimulated with PAMPS or DAMPS via TLR or ATP receptors. In the presence of LPS and IFN-γ, microglial cells polarize to M1 phenotype and produce pro-inflammatory cytokines/mediators including IL-1β, IL-6, TNF-α, CCL2, ROS, and NO. In contrast, IL-4 and IL-13 induce alternative activation of microglia to M2 (‘M2a’) phenotype which down-regulates M1 functions by anti-inflammatory cytokine, IL-10.

Microglial cells express several macrophage-associated markers, such as CD11b, CD14, CX3C chemokine receptor 1 (CX3CR1, fractalkine receptor), ionized calcium-binding adaptor molecule-1 (Iba-1), and F4/80 (also known as EMR1) [10,13]. Unlike neurons, astrocytes, or oligodendrocytes, microglial cells are shown to be derived from hematopoietic stem cells in the yolk sac and act as primary responding cells for pathogen infections and injuries in the CNS [10,13]. It is likely that microglia contribute to maintenance of tissue homeostasis and act as sentinels of infection and injury to participate in both innate and adaptive immune responses in the CNS [10,11,13]. Like macrophages, microglial cells polarize to M1 phenotype by stimulation with LPS and IFN-γ and can produce pro-inflammatory cytokines/mediators such as IL-1β, IL-6, TNF-α, CCL2, ROS, and NO [10,13]. On the other hand, it has been reported that intracerebral injection of IL-4/IL-13 up-regulates expressions of Arg-1, Ym1, and CD36 in activated microglia and reduces TNF-α levels in the CNS of mice [24]. Thus, IL-4 and IL-13 can induce alternative activation and polarization of M2 (‘M2a’) microglia which express M2 markers and anti-inflammatory cytokine, IL-10 [8,13,18]. Based on these evidences, there is a possibility that M1/M2 polarization of microglia plays an important role in controlling the balance between promotion and resolution in neuroinflammation in the CNS (Figure 1).

The concept of differential M1 and M2 polarization of macrophages was presumed from the classical dichotomous activation program of Th1 and Th2 cells. However, it should be noted that the views of classifying macrophages or microglial cells in either M1 or M2 polarized state might be an oversimplification, because macrophages and microglial cells show a high-degree of diversity and plasticity [20,25]. Consequently, further functional studies are required to understand the immunological properties of macrophages and microglial cells under normal and pathological circumstances with regard to the M1/M2 concept.

## 3. Schizophrenia

Schizophrenia affects approximately 1% of the population and the onset of the disorder is typically in late adolescence or early adulthood [26]. The symptoms of schizophrenia are commonly divided into three major categories, positive, negative, and cognitive [9]. Impaired social cognition is a defining feature of schizophrenia, although it is observed in many psychiatric disorders [27]. Social cognition is considered to be significantly involved in quality of social life and to be influenced by the course of disorder such as relapse [27]. There has been a growing interest in the physiological mechanism of relapse of schizophrenia [28], because prevention of relapse is one of the most important tasks of therapy for this disorder. Several hypotheses of schizophrenia based on the abnormalities of dopamine and glutamate neurotransmissions were provided; dopamine hyperactivity in nucleus accumbens induced by hypofunction of glutamate receptors in ventral tegmental area is hypothesized to be associated with positive symptoms, while hypoactivation of dopamine and glutamate neurons in prefrontal cortex is considered to contribute to negative symptoms [9]. Although several recent studies suggest involvement of neuroinflammation in schizophrenia [12,14], few of these hypotheses provide an effective explanation on the mechanisms of relapse and remission.

Recent studies have demonstrated the abnormalities of potassium ion channels expressed on astrocytes in the hippocampus where neuronal hyperactivation plays an important role in symptoms of patients with temporal lobe epilepsy (TLE) [29]. Impaired potassium ion buffering and dysfunction of a water channel, aquaporin 4 (AQP4) increase the resting membrane potential of neurons, resulting in neuronal hyperactivation and subsequent neuronal death [30,31]. After the neuronal hyperactivation, ATP derived from the dead neurons can induce polarization of microglia to a M1 phenotype (M1 microglia) [10,32]. Similar abnormalities or alterations may be induced in the brain of schizophrenia. It has been reported that individuals with an At Risk Mental State (ARMS) and patients with the first-episode or chronic schizophrenia have lower volumes of insula, inferior frontal gyrus, and hippocampus [33,34], suggesting that these brain areas play an important role in initiation of schizophrenia. In the brain of schizophrenia patients, a positron emission tomography (PET) study has indicated a significant increase in binding potential of *(R)*-[^11^C]PK11195, a parameter of microglia activation in total gray matter [34]. The binding potential of *(R)*-[^11^C]PK11195 is clearly higher in the hippocampus of schizophrenia patients than in that of healthy control [35,36]. Furthermore, the levels of IL-1β, IL-6, IL-8, and TNF-α in the cerebrospinal fluid (CSF) or peripheral blood are significantly higher in schizophrenia patients as compared with healthy volunteers [37,38,39,40]. In addition, there is a possibility that these cytokines produced in the brain are leaking to the periphery, or are produced by peripheral immune cells [14]. Based on these results, it is suggested that M1 polarization of microglia is induced in the insula, inferior frontal gyrus, and hippocampus of the patients with schizophrenia and that pro-inflammatory cytokine levels in the CSF and blood partly reflect polarization of M1 microglia in the CNS.

It has been demonstrated that TNF-α can induce glutamate release from astrocytes and that glutamate up-regulates TNF-α production by microglia [41,42]. Glutamate can induce dysfunction of oligodendrocytes via glutamate receptors [43,44]. Schizophrenia patients show abnormalities of myelination detected with magnetic resonance imaging (MRI) scans and post-mortem analysis of oligodendrocyte proteins [45]. Functional MRI (fMRI) studies have revealed a significant failure of reciprocal influence between insula and dorsolateral prefrontal cortex (DLPFC) in schizophrenia [46]. These observations suggest that M1 microglia can promote glutamate release to induce dysfunction of oligodendrocytes, resulting in abnormalities of neural network in the brain of patients with schizophrenia.

A significant increase in IL-10 levels has been reported in the serum of schizophrenia patients [47]. Recently, relatively higher amounts of IL-10 and IL-13 have been found in the CSF and blood from patients with schizophrenia [48]. From these results, it is highly probable that anti-inflammatory responses mediated by IL-10 and IL-13, which reflect M2 polarization of microglia, occur in the CNS of schizophrenia patients. On the other hand, it has been documented that concomitant treatment with a cyclooxygenase-2 (COX-2) inhibitor, celecoxib and an antipsychotic (risperidone or amisulpride) significantly improves Positive and Negative Syndrome Scale (PANSS) scores of schizophrenia patients in double-blind, placebo-controlled clinical trials [49,50,51]. Recent animal studies have revealed that treatment with celecoxib reduces the number of activated microglia and IL-1β levels in the brain of rat injected with LPS [52]. Celecoxib is known to inhibit production of IL-1β and TNF-α by macrophages stimulated with LPS and IFN-γ [53]. Taken together, it is strongly suggested that celecoxib inhibits production of pro-inflammatory cytokines by M1 microglia and potentiates therapeutic effects of antipsychotics in schizophrenia patients. Similarly, IL-10 from M2 microglia may contribute to remission in schizophrenia by inhibiting pro-inflammatory cytokine production in M1 microglia.

Although IL-4 and IL-13 are thought to be key molecules to skew M2 polarization of macrophages and microglia [54], CCL2 and IL-6 produced by M1 microglia may be the other important molecules to induce M2 polarization of microglia because CCL2 and IL-6 are produced in neurodegenerative and neuroinflammatory conditions and can induce M2 polarization of macrophages [22]. IL-10 seems to be predominantly involved in anti-inflammatory functions of M2 microglia, and lipid mediators such as resolvin D1 and lipoxin A4 may also contribute to anti-inflammatory responses because these lipid mediators are produced by M2 macrophages and can induce anti-inflammatory conditions [55,56].

**Figure 2:**
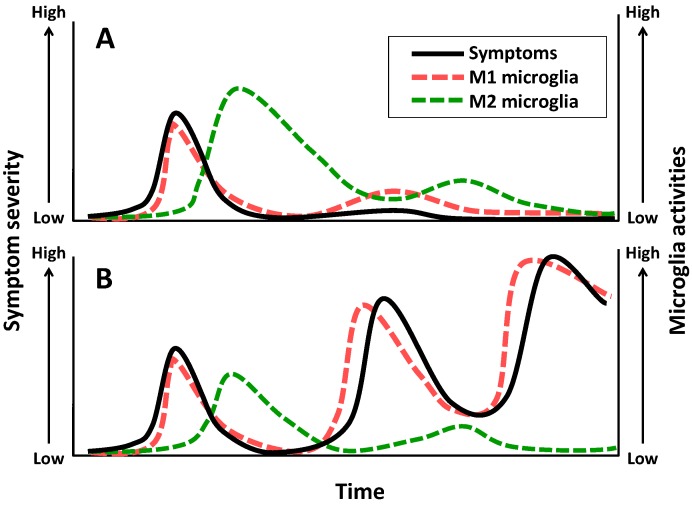
Hypothetical model of relationship between M1/M2 microglia activities and symptom severity in schizophrenia. (**A**) In the early stage of schizophrenia, symptoms may be followed by microglial M1 polarization which is induced by neuronal hyperactivation in insula, inferior frontal gyrus, and hippocampus, possible initiating brain regions of the disorder. M1 microglia can produce pro-inflammatory cytokines and remove the damaged nerve fibers by phagocytosis, whereas M2 microglia down-regulate M1 microglial function and restore tissue homeostasis with consequent attenuation of symptoms. (**B**) If M2 polarization of microglia is insufficient, M1 microglial functions are maintained and induce neural network dysfunctions continuously. Symptom severity may gradually become high according to the frequency of M1 polarization.

Based on the immunoregulatory functions of M1 and M2 microglia, there is a possibility that relapse of schizophrenia is associated with pro-inflammatory M1 polarization of microglia, whereas remission is related to anti-inflammatory M2 polarization of microglia that can inhibit M1 microglial functions and maintain tissue homeostasis. If the microenvironment of the brain in schizophrenia patients is insufficient to skew M2 polarization of microglia, M1 microglia can continuously induce abnormalities of oligodendrocytes in any brain regions, gradually impairing neural network functions required for social life such as social cognition (Figure 2). Our view supports a hypothesis by Meyer *et al.* suggesting that enhancement of anti-inflammatory activity attenuates pro-inflammatory responses and schizophrenia symptoms, whereas reduced potency of anti-inflammatory responses exacerbates the symptoms accompanied with elevation of pro-inflammatory activity [12]. There has been evidence that mitochondrial dysfunction is involved in psychiatric disorders [57,58,59]. A recent study has demonstrated that mitochondrial dysfunction inhibits microglial activation induced by IL-4 but not LPS [60]. Therefore, mitochondria may play a key role in M2 polarization of microglia. Impairment of social cognition and abnormalities of microglial function in the brain have been demonstrated in patients with autism [61,62], suggesting common brain alterations in schizophrenia and autism.

## 4. Major Depressive Disorder

Major depressive disorder is characterized by two major symptoms, depressed mood and loss of interest/pleasure feelings [9]. The monoamine hypothesis of depression is widely accepted; depressed mood is predominantly caused by the reduced activity of 5-hydroxytryptamine (5-HT) neurons projecting to prefrontal cortex; hypoactivation of dopamine neurons in nucleus accumbens is involved in loss of interest and pleasure; in addition, noradrenaline neurons seem to be implicated in these symptoms [9].

fMRI studies in patients with major depressive disorder have demonstrated hyperactivation of neurons in neural circuitry of mood including amygdala and hippocampus [63]. The prefrontal cortex including DLPFC regulates neural circuitry of mood, affecting stress responses [64,65]. Hypoactivation and loss of synapses in DLPFC have been reported in patients with major depressive disorder [66]. Based on these findings, we speculate as follows; dysfunction of potassium ion channels and AQP4 expressed on astrocytes induces hyperactivation of neural circuitry of mood and M1 polarization of microglia; glutamate released from M1 microglia and activated astrocytes causes dysfunction of nerve fibers between prefrontal cortex and the neural circuitry, resulting in dysregulation and hypoactivation of the prefrontal cortex where 5-HT neurons project. Our view supports the monoamine hypothesis of depression suggesting relationship between depressed mood and hypoactivation of 5-HT neurons in the prefrontal cortex.

**Figure 3:**
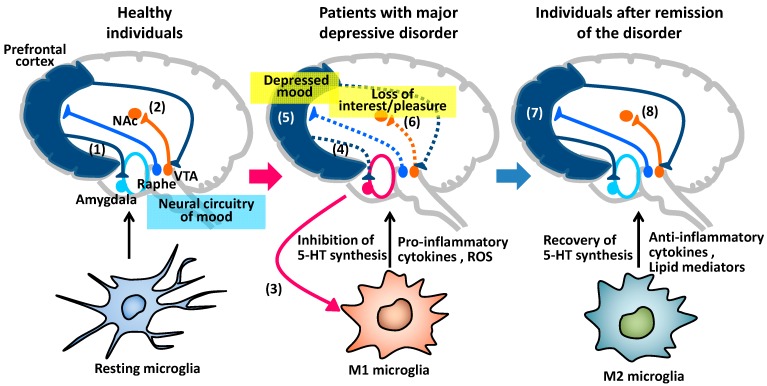
Possible roles of M1/M2 microglia in neural network functions, activities of monoamine neurons, and symptoms in major depressive disorder. In healthy individuals, prefrontal cortex regulates neural circuitry of mood including amygdala and dopamine neurons projecting from VTA (ventral tegmental area) to NAc (nucleus accumbens) (1, 2). In patients with major depressive disorder, hyperactivation of neural circuitry induces M1 polarization of microglia (3), resulting in dysfunction of nerve fibers between prefrontal cortex and the neural circuitry (4) and hypoactivation of 5-HT neurons projecting from raphe nucleus to prefrontal cortex (5). Dysfunction of prefrontal cortex can reduce activity of dopamine neurons projecting from VTA to NAc (6). Hypoactivation of prefrontal cortex and NAc are associated with depressed mood and loss of interest/pleasure, respectively. M2 microglia restore homeostasis of nerve fibers and 5-HT biosynthesis, recovering dysfunction of prefrontal cortex and NAc (7, 8).

There is another possible mechanism of reduction of 5-HT neuron activity. It has been reported that IL-1β and IL-6 potentiate the metabolic pathways from tryptophan to kynurenic acid and quinolinic acid [67,68], suggesting that M1 microglia can decrease 5-HT production. It is presumed that dysfunction of the prefrontal cortex induces dopamine hypoactivity of nucleus accumbens because the prefrontal cortex regulates the ventral tegmental area from which dopamine neurons project to the nucleus accumbens [65,69]. On the other hand, if M2 microglia restore homeostasis of oligodendrocytes and 5-HT biosynthesis, dysfunction of prefrontal cortex (depressed mood) would be improved, followed by attenuation of nucleus accumbens hypoactivation (loss of interest and pleasure) (Figure 3).

Several double-blind, placebo-controlled clinical studies have revealed that celecoxib in combination with an antidepressant significantly improves Hamilton Rating Scale for Depression (HAMD) scores in patients with major depressive disorder [70,71], suggesting that neuroinflammation is related to major depressive disorder as well as schizophrenia. These findings support our hypothesis that M1 and M2 phenotypes of microglia are closely related to relapse and remission, respectively in major depressive disorder. Furthermore, our hypothesis can explain the relationship between neuroinflammation, hypoactivation of monoamine neurons, and that of brain regions associated with two major symptoms. However, there is still an unresolved question; the patients with major depressive disorder show dopamine hypoactivity in nucleus accumbens, whereas schizophrenia patients have dopamine hyperactivity in the same brain region. Thus, it is necessary to identify the neural circuitries of social cognition, positive, and negative symptoms in schizophrenia, and to clarify the differences between neural networks of major depressive disorder and schizophrenia.

## 5. Vascular Depression

It has been reported that depressive symptoms appear after stroke (post-stroke depression), and that elderly patients with major depressive disorder frequently show white matter hyperintensities and silent infarction of gray matter in the brain (MRI-defined vascular depression) [72,73,74]. Post-stroke depression and MRI-defined vascular depression are termed vascular depression by Alexopoulos *et al.* [75], although it is controversial whether these depressions are in the same category. In contrast to major depressive disorder without any physical causes, neuroimaging analyses as well as symptoms are important to characterize vascular depression. Antidepressants provide unstable remission and low response rate to vascular depression [75,76,77]. Vascular depression is presumed to be associated with neurocognitive disorders (dementia), because cerebrovascular lesions are one of significant etiological factors of neurocognitive disorders, and major depressive disorder is considered to be one of risk factors for neurocognitive disorders, frequently appearing as a prodrome of the disorders [78,79,80].

It is presumed that there is a relationship between cerebrovascular lesions and aging, hypertension, dyslipidemia, atherosclerosis, and diabetes [75]. Abnormalities in capillary endothelial cells of the brain induced by these events can activate platelets and neutrophils [81]. The activated platelets and neutrophils in inflamed capillaries produce various pro-inflammatory cytokines/chemokines and induce recruitment of monocytes/macrophages [81]. M1 polarization of recruited macrophages is induced by pro-inflammatory cytokines and M1 macrophages remove injured vascular endothelial cells by phagocytosis [81,82]. M1 macrophages may induce long-term inflammation and destruction of capillary venules if M2 polarization is insufficient.

Vascular endothelial cells in the brain capillary are known to produce platelet-derived growth factor (PDGF) which can induce production of vascular endothelial growth factor (VEGF) and angiopoietin by PDGF receptor β-expressing pericytes and astrocytes [83,84,85,86]. On the other hand, pericytes can produce CXC-chemokine ligand (CXCL) 12, while CXCR4 (CXCL12 receptor) is expressed on vascular endothelial cells and astrocytes [87,88]. From these findings, it is suggested that the interaction among vascular endothelial cells, pericytes, and astrocytes plays an important role in maintenance of BBB functions for sufficient supply of oxygen and nutrients to neurons [89,90]. Because pro-inflammatory cytokines and ROS from M1 macrophages can induce inflammation and tissue damage in vascular endothelium, it is probable that M1 macrophages trigger impairment of BBB functions and subsequent neuronal death in the brain as seen in neuroimaging analyses. Our view is supported by Zlokovic suggesting that oxidative damage of vascular endothelium occurs before neuronal deposition in Alzheimer’s disease [80].

The neuronal death can induce M1 polarization of microglia in the brain and pro-inflammatory functions of M1 microglia may result in nerve fiber and neural network abnormalities related to mood and neurocognition. The location of stroke lesions has been reported not to be the exclusive etiological factor in post-stroke depression [91]. Thus, in agreement with Taylor *et al.* [76], we hypothesize that symptoms of vascular depression are associated with not only brain lesions resulted from the impaired BBB but also abnormalities of neural network in the brain. The impaired BBB function-induced dysfunction of neural network in the brain may occur in elderly patients of late-onset with vascular depression, because it has been reported that the severity of deep white matter low density shows a significantly positive correlation with age at onset in major depressive disorder, suggesting that vascular changes are more severe in the elderly patients [92]. To better understand how patients with vascular depression develop neurocognitive disorders, it is noteworthy to study the interaction between prefrontal cortex, neural circuitry of mood, and that of neurocognition.

## 6. Chronic Pain

Chronic pain is classified as a psychiatric disorder and pain itself is a physically primitive but complicated perception. Pain experience is affected by psychological factors such as social learning, cognition, and other psychiatric disorders [93,94]. Pro-inflammatory cytokines such as IL-1β and TNF-α are significantly increased in the CSF and blood in patients with chronic neuropathic pain [95,96], suggesting a role of M1-mediated neuroinflammation in this disorder.

The possible neural pathways of cognitive pain modulation have been proposed by Tracey and colleagues [94,97]. Cognitive modulations of pain are referred to activation of brain areas such as DLPFC, ventrolateral prefrontal cortex (VLPFC), and anterior cingulate cortex (ACC), which modulate activation of pain-associated circuitry including somatosensory cortex, insula, and thalamus where ascending nociceptive signals input. fMRI studies revealed that patients with chronic back pain show decrease in prefrontal and thalamic gray matter density, which are likely due to hyperactivation of these brain areas [98], while activations in DLPFC have been found in studies on placebo-induced analgesia in humans [99]. That is, it is plausible that M1 polarization of microglia is induced in the thalamus, one of the possible initiating regions of patients with chronic pain, resulting in abnormalities of nerve fibers between the regions for cognitive pain modulation and pain-associated circuitry, which induce dysregulation and hypoactivation of these regions.

It has been suggested that the prefrontal cortex plays an important role in “keeping pain out of mind” in chronic pain [100], and that perception of pain is sensitive to the beliefs that someone has about pain [94]. Therefore, we speculate that “pseudo-experience of pain”, which is not based on activity of the pain-associated circuitry, occurs in patients with chronic pain. Pseudo-experience of pain may be a kind of cognitive distortion as seen in major depressive disorder, because cognitive distortions are associated with hypoactivation of the prefrontal cortex. A recent report has proposed that there are possible common brain alterations in chronic migraines and mood disorders such as major depressive disorder [101]. The conceptualization of migraine has evolved from a vascular disorder to a neurovascular disorder and currently to a brain disorder, primarily a neural network disorder [101]. Taken together, symptoms of major depressive disorder, vascular depression, chronic pain, and migraine may be induced by common mechanisms, dysregulation of the prefrontal cortex for the lower brain circuitries of mood and pain. Our hypothesis is supported by the views that mood and pain experience influence each other, and that mood can change pain thresholds [94,97].

## 7. Molecules to Skew M2 Polarization of Microglia

### 7.1. Endocannabinoids and Cannabinoid Receptors

The endocannabinoid levels in the CSF have been shown to be negatively correlated with symptom severity of schizophrenia [102]. Cannabidiol, an inhibitor of endocannabinoid-degrading enzyme, has been reported to significantly improve PANSS scores in schizophrenia patients in a double-blind study [103]. Blaas has found that dronabinol, an agonist for cannabinoid receptors improves depressed mood when treated alone or in combination with antidepressants [104]. Based on these evidences, we discuss possible roles of endocannabinoids and their receptors in M1/M2 microglial polarization in psychiatric disorders and diseases.

There has been a growing interest in the roles of cannabinoid receptors in neuroinflammation in the CNS. 2-Arachidonoyl-glycerol (2-AG), one of the endogenous cannabinoids, is biosynthesized in various types of cells including macrophages and microglial cells, and binds to two distinct cannabinoid receptors (CB_1_ and CB_2_) [105,106]. The CB_1_ receptor is expressed in various tissues constitutively and higher expressions are found in neural cells. On the other hand, the CB_2_ receptor is inducible and expressed in immune cells predominantly [105,107]. CB_1_ agonist promotes pro-inflammatory responses of macrophages through ROS production, which is negatively regulated by CB_2_ through Rap1 activation [108]. Furthermore, CB_1_ agonists induce biosynthesis of ceramide by sphingomyelinase [109]. Thus, the CB_1_ appears to contribute to polarization and maintenance of M1 macrophages and microglia. On the other hand, agonistic stimulation by 2-AG can induce internalization and down-regulation of CB_1_ [110]. Taken together, it is presumed that 2-AG induces down-regulation of CB_1_ and up-regulation of CB_2_ conversely. Thus, there seems to be an inhibitory interaction between CB_1_ and CB_2_ functions.

**Figure 4:**
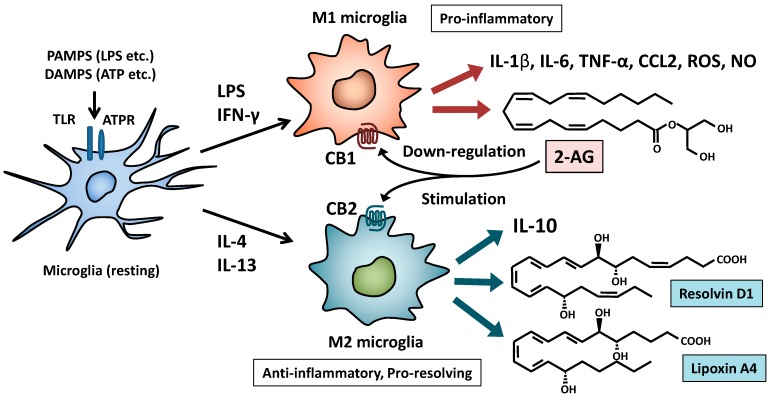
Possible roles of the cannabinoid receptors in M1/M2 polarization of microglia. 2-AG released from M1 microglia promotes production of pro-inflammatory cytokines and mediators by M1 microglia via CB_1_ and then induces down-regulation of CB_1_. On the other hand, 2-AG stimulates M2 polarization of microglia via CB_2_. Subsequently, M2 microglia can produce IL-10 and anti-inflammatory/pro-resolving lipid mediators (resolvin D1 and lipoxin A4).

Klein and colleagues have reported that Δ^9^-tetrahydrocannabinol (THC, an agonist for CB_1_ and CB_2_) increases mRNA expression of GATA binding protein 3 (GATA3) and IL-4 production in the spleen of *Legionella pneumophila-*infected mice, but THC shows no effects in CB_2_ knockout mice [111,112]. These findings suggest that the CB_2_ plays an essential role in differentiation of Th2 cells or M2 polarization of macrophages in bacterial infections. It has been reported that IL-4 significantly decreases inducible NO synthase (iNOS) expression and NO release via PPAR-γ in pro-inflammatory cytokine-treated CNS glial cells [113]. The CB_2_ signaling can induce *de novo* synthesis of ceramide via serine-palmitoyltransferase [114]. Ceramide is metabolized by ceramidase to the long-chain fatty acids (LCFAs) and sphingosine [115]. LCFAs activate PPAR-γ, while sphingosine-1-phospahe (S1P) has anti-apoptotic or cytoprotective effects and up-regulates IL-4 production in CD4 T cells [116,117,118]. Based on these results, it is strongly suggested that 2-AG-CB_1_ axis contributes to polarization and maintenance of M1 microglia, while 2-AG-CB_2_ axis acts as a switch from M1 to M2 polarization of microglia (Figure 4). CB_2_ agonists are known to induce phosphorylation of AMP-activated protein kinase (AMPK), suggesting that the CB_2_ plays an important role in AMPK-mediated anti-oxidative and cytoprotective effects [119,120,121]. Furthermore, 2-AG is reported to activate PPAR-γ in M2 macrophages [122]. Thus, AMPK may be one of key signal molecules for the switch to M2 polarization. Besides endocannabinoids, adiponectin and ghrelin can induce down-stream signal transduction of their receptors via AMPK and therefore these molecules may be involved in skewing M2 polarization of microglia [123,124].

### 7.2. Anti-Inflammatory and Pro-Resolving Lipid Mediators

The analyses of cellular and molecular mechanisms of the resolution of inflammation have revealed the key roles of anti-inflammatory and pro-resolving lipid mediators such as lipoxin A4, resolvin D1, resolvin E1, and protectin D1 [125]. These mediators are mainly biosynthesized from docosahexaenoic acid (DHA) or arachidonic acid by 15-lipoxygenase [125]. Resolvin D1 and lipoxin A4 are known to exhibit an agonistic activity at GPR32 and lipoxin A4 receptor/*N*‑formyl peptide receptor 2 (ALX/FPR2) [126]. Resolvin D1 up-regulates the levels of micro-RNAs (miR-208a and miR-219) and enhances IL-10 production by peritoneal exudate macrophages in zymosan-induced peritonitis in ALX/FPR2 transgenic mice [126]. Furthermore, it has been reported that resolvin D1 and DHA can induce M2 polarization of macrophages [127] and that ALX/FPR2 is expressed on macrophages and microglia [128]. A double-blind, placebo-controlled clinical studies revealed that the transition rate to psychotic disorder is significant lower in ARMS individuals received with capsules containing DHA and eicosapentaenoic acid (EPA) as compared with placebo-treated controls [129]. Furthermore, ethyl-EPA in combination with antipsychotics has been reported to improve PANSS scores significantly in schizophrenia patients [130]. From these results, it is strongly suggested that anti-inflammatory and pro-resolving lipid mediators such as resolvin D1 and lipoxin A4 play an important role in polarization and maintenance of M2 microglia (Figure 4).

## 8. Conclusions

In this review, we provide a hypothesis that M1 and M2 phenotypes of microglia are closely related to relapse and remission, respectively, in psychiatric disorders and diseases. M1 polarization of microglia seems to induce dysfunction of the neural network in the CNS. Specifically, it is presumed that M1 microglia-induced dysregulation of prefrontal cortex for the neural circuitries of mood and pain results in symptoms of major depressive disorder, vascular depression, chronic pain, and migraine. M2 polarized microglia presumably attenuate M1 microglia-mediated neuroinflammation by production of anti-inflammatory cytokine, IL-10. On the other hand, further studies on M2 microglial functions are necessary to understand their exact roles in neuroinflammation, because M2 macrophages seem to induce Th2-type inflammatory conditions [131,132]. Since endocannabinoids, adiponectin, ghrelin, or anti-inflammatory/pro-resolving lipid mediators appear to skew M2 polarization of microglia, modulation of these molecules may afford favorable approaches for treatment of vascular depression to reduce a risk for neurocognitive disorders. Consequently, the molecules skewing M2 phenotype of microglia may provide a beneficial therapy to attenuate relapse of psychiatric disorders and diseases.

## References

[1] Dutta R., Trapp B.D. (2007). Pathogenesis of axonal and neuronal damage in multiple sclerosis. Nerology.

[2] Lasmann H., Bruck W., Lucchinetti C.F. (2007). The immunopathology of multiple sclerosis: an overview. Brain Pathol..

[3] Frohman E.M., Racke M.K., Raine C.S. (2006). Multiple sclerosis—The plaque and its pathogenesis. N. Engl. J. Med..

[4] Pelfrey C.M., Tranquill L.R., Vogt A.B., McFarland H.F. (1996). T cell response to two immunedominant proteolipid protein (PLP) peptides in multiple sclerosis patients and healthy controls. Mut. Scler..

[5] McFarland H.F., Martin R. (2007). Multiple sclerosis: A complicated picture of autoimmunity. Nat. Immunol..

[6] Steinman L. (2010). Mixed results with modulation of TH-17cells in human autoimmune diseases. Nat. Immunol..

[7] Steinman L. (2009). A molecular trio in relapse and remission in multiple sclerosis. Nat. Rev. Immuno..

[8] Mikiat J., Doubourdieu-Cassagno N., Deloire M.S., Vekrie A., Biran M., Raffard G., Brochet B., Canron M.H., Franconi J.M., Boiziau C. (2011). Amelioration of clinical status by M2 activated monocyte administration. Mult. Scler..

[9] Stahl S.M. (2008). Stahl’s Essential Psychopharmacology: Neuroscientific Basis and Practical Applications.

[10] Kettenmann H., Hanisch U.K., Noda M., Verhratskym A. (2011). Physiology of microglia. Physiol. Rev..

[11] Ransohoff R.M., Perry V.H. (2009). Microgilal physiology: Unique stimuli, specialized resonses. Annu. Rev. Immunol..

[12] Meyer U., Schwarz M.J., Müller N. (2011). Inflammatory processes in schizophrenia: A promising neuroimmunological target for the treatment of negative/cognitive symptoms and beyond. Pharmacol. Ther..

[13] Saijo K., Glass C.K. (2011). Microglial cell origin and phenotypes in health and disease. Nat Rev. Immunol..

[14] Beumer W., Gibney S.M., Drexhage R.C., Pont-Lezica L., Doorduin J., Klein H.C., Steiner J., Connor T.J., Harkin A., Versnel M.A. (2012). The immune theory of psychiatric diseases: a key role for activated microglia and circulating monocytes. J. Leukoc. Biol..

[15] Chow A., Brown B.D., Merrad M. (2011). Studying the mononuclear phagocyte system in the molecular age. Nat. Rev. Immunol..

[16] Geissmann F., Jung S., Littman D.R. (2003). Blood monocytes consist of two principal subsets with distinct migratory properties. Immunity.

[17] Geissmann F., Manz M.G., Jung S., Sieweke M.H., Merad M., Leym K. (2010). Development of monocytes, macrophages, and dendritic cells. Science.

[18] Mosser D.M., Edwards J.P. (2008). Exploring the full spectrum of macrophage activation. Nat. Rev. Immunol..

[19] Gordon S., Martinez F.O. (2010). Alternative activation of macrophages: Mechanism and functions. Immunity.

[20] Prinz M., Priller J. (2014). Microglia and brain macrophages in the molecular age: From origin to neuropsychiatric disease. Nat. Rev. Neurosci..

[21] Wynn T.A., Chawla A., Pollard J.W. (2013). Macrophage biology in development, homeostasis, and disease. Nature.

[22] Roca H., Varsos Z.S., Sud S., Craig M.J., Ying C., Pienta K.J. (2009). CCL2 and interleukin-6 promote survival of human CD11b+ peripheral blood mononuclear cells and induce M2-type macrophage polarization. J. Biol. Chem..

[23] Lawrence T., Natoli G. (2011). Transcriptinal regulation of macrophage polarization enabling diversity with identity. Nat. Rev. Immunol..

[24] Kawahara K., Suenobu M., Yoshida A., Koga K., Hyodo A., Ohtsuka H., Kuniyasu A., Tamamaki N., Sugimoto Y., Nakayama H. (2012). Intracerebral microinjection of interleukin-4/interleukin-13 reduces β-amyloid accumulation in the ipsilateral side and improves cognitive deficits in young amyloid precursor protein 23 mice. Neuroscience.

[25] Mittelbronn M. (2014). The M1/M2 immune polarization concept in microglia: A fair transfer?. Neuroimmunol. Neuroinflamm..

[26] Tandon R., Keshavan M.S., Nasrallah H.A. (2008). Schizophrenia, “just the facts” what we know in 2008. 2. Epidemiology and etiology. Schizophr. Res..

[27] Couture S.M., Penn1 D.L., Roberts D.L. (2006). The functional significance of social cognition in schizophrenia: a review. Schizophr. Bull..

[28] Leucht S., Tardy M., Komossa K., Heres S., Kissling W., Salanti G., Davis J.M. (2012). Antipsychotic drugs *versus* placebo for relapse prevention in schizophrenia: A systematic review and meta-analysis. Lancet.

[29] Hinterkeuser S., Schroder W., Hanger G., Seifert G., Blumcke I., Elger C.E., Schramm J., Steinhauser C. (2000). Astrocytes in the hippocampus of patients with temporal lobe epilepsy display changes in potassium conductances. Eur. J. Neurosci..

[30] Seifert G., Carmignoto G., Steinhäuser C. (2010). Astrocyte dysfunction in epilepsy. Brain Res. Rev..

[31] Wetherington J., Serrano G., Dingleline R. (2008). Astrocytes in the epileptic brain. Neuron.

[32] David S., Kroner A. (2011). Repertoire of microglial and macrophage responses after spinal cord injury. Nat. Rev. Neurosci..

[33] Borgwardt S.J., Riecher-Rössler A., Dazzan P., Chitnis X., Aston J., Drewe M., Gschwandtner U., Haller S., Pflüger M., Rechsteiner E. (2007). Regional gray matter volume abnormalities in the At Risk Mental State. Biol. Psychiatry..

[34] Chan R.C.K., Di X., McAlonan G.M., Gong Q. (2011). Brain anatomical abnormalities in high-risk individuals, first-episode, and chronic schizophrenia: An activation likelihood estimation meta-analysis of illness progression. Schizophr. Bull..

[35] Van Berckel B.N., Bossong M.G., Boellaard R., Kloet R., Schuitemaker A., Caspers E., Luurtsema G., Windhorst A.D., Cahn W., Lammertsma A.A. (2008). Microglia activation in recent-onset schizophrenia: a quantitative *(R)*-[11C]PK11195 positron emission tomography study. Biol. Psychiatry.

[36] Doorduin J., de Vries E.F.J., Willemsen A.T.M., de Groot J.C., Dierckx R.A., Klein H.C. (2009). Neuroinflammation in schizophrenia-related psychosis: a PET study. J. Nucl. Med..

[37] Barak V., Barak Y., Levine J., Nisman B., Roisman I. (1995). Changes in interleukin-1β and soluble interleukin-2 receptor levels in CSF and serum of schizophrenic patients. J. Basic. Clin. Physiol. Pharmacol..

[38] Theodoropoulou S., Spanakos G., Baxevanis C.N., Economou M., Gritzapis A.D., Papamichail M.P., Stefanis C.N. (2001). Cytokine serum levels, autologous mixed lymphocyte reaction and surface marker analysis in never medicated and chronically medicated schizophrenic patients. Schizophr. Res..

[39] Zhang X.Y., Zhou D.F., Zhang P.Y., Wu G.Y., Cao L.Y., Shen Y.C. (2002). Elevated interleukin-2, interleukin-6 and interleukin-8 serum levels in neuroleptic-free schizophrenia: Association with psychopathology. Schizophr. Res..

[40] Garver D.L., Tamas R.L., Holcomb J.A. (2003). Elevated interleukin-6 in the cerebrospinal fluid of a previously delineated schizophrenia subtype. Neuropsychopharmacology.

[41] Bezzi P., Domercq M., Brambilla L., Galli R., Schols D., de Clercq E., Vescovi A., Bagetta G., Kollias G., Meldolesi J. (2001). CXCR4-activated astrocyte glutamate release via TNFα: amplification by microglia triggers neurotoxicity. Nat. Neurosci..

[42] Taylor D.L., Jones F., Kubota E.S.F.C.S., Pocock J.M. (2005). Stimulation of microglial metabotropic glutamate receptor mGlu2 triggers tumor necrosis factor α-induced neurotoxicity in concert with microglial-derived Fas ligand. J. Neurosci..

[43] Patneau D.K., Wright P.W., Winters C., Mayer M.L., Gallo V. (1994). Glial cells of the oligodendrocyte lineage express both kainate- and AMPA-preferring subtypes of glutamate receptor. Neuron.

[44] Káradóttir R., Attwell D. (2006). Neurotransmitter receptors in the life and death of oligodendrocytes. Neuroscience.

[45] Flynn S.W., Lang D.J., Mackay A.L., Goghari V., Vavasour I.M., Whittall K.P., Smith G.N., Arango V., Mann J.J., Dwork A.J. (2003). Abnormalities of myelination in schizophrenia detected *in vivo* with MRI, and post-mortem with analysis of oligodendrocyte proteins. Mol. Psychiatry.

[46] Palaniyappan L., Simmonite M., White T.P., Liddle E.B., Liddle P.F. (2013). Neural primacy of the salience processing system in schizophrenia. Neuron.

[47] Maes M., Chiavetto L.B., Bignotti S., Tura G.J.B., Oioli R., Boin F., Kenis G., Bosmans E., de Jongh R., Altamura C.A. (2002). Increased serum interkeukin-8 and interleukin-10 in schizophrenic patients resistant to treatment with neuroleptics and the stimulatory effects of clozapine on leukemia inhibitory factor receptor. Schizophr. Res..

[48] Maxeiner H.G., Schneider E.M., Kurfiss S.T., Brettschneider J., Tumani H., Bechter K. (2014). Cerebrospinal fluid and serum cytokine profiling to detect immune control of infectious and inflammatory neurological and psychiatric diseases. Cytokine.

[49] Müller N., Riedel M., Scheppach C., Brandstätter B., Sokullu S., Krampe K., Ulmschneider M., Engel R.R., Möller H.J., Schwarz M.J. (2002). Beneficial antipsychotic effects of celecoxib add-on therapy compared to risperidone alone in schizophrenia. Am. J. Psychiatry.

[50] Müller M., Krause D., Dehning S., Musil R., Schennach-Wolff R., Obermeier M, Möller H.J., Klauss V., Schwarz M.J., Riedel M. (2010). Celecoxib treatment in an early stage of schizophrenia: results of a randomized, double-blind, placebo-controlled trial of celecoxib augmentation of amisulpride treatment. Schizophr. Res..

[51] Zhang Y., Chun Chen D., Long Tan Y., Zhou D.F. (2006). A doubleblind, placebo-controlled trial of celecoxib added to risperidone in first-episode and drug-naive patients with schizophrenia. Eur. Arch. Psychiatry Clin. Neurosci..

[52] Kaizaki A., Tien L.T., Pang Y., Cai Z., Tanaka S., Numazawa S., Bhatt A.J., Fan L.W. (2013). Celecoxib reduces brain dopaminergic neuronaldysfunction, and improves sensorimotor behavioral performance in neonatal rats exposed to systemic lipopolysaccharide. J. Neuroinflamm..

[53] Hung Y., Liu J., Wang L., Zhang W., Zhu X. (2005). Neuroprotective effects of cyclooxygenase-2 inhibitor celecoxib against toxicity of LPS-stimulated macrophages toward motor neurons. Acta Paharmacol. Sin..

[54] Cherry J.D., Olschowka J.A., O’Banion M.K. (2014). Neuroinflammation and M2 microglia: The good, the bad, and the inflamed. J. Neuroinflamm..

[55] Ortega-Gomez A., Perretti M., Soehnlein O. (2013). Resolution of inflammation: An integrated view. EMBO Mol. Med..

[56] Alessandri A.L., Sousa L.P., Lucas C.D., Rossi A.G., Pinho V., Teixeira M.M. (2013). Resolution of inflammation: Mechanisms and opportunity for drug development. Pharmacol. Ther..

[57] Kato T., Kato N. (2000). Mitochondrial dysfunction in bipolar disorder. Bipolar Disord..

[58] Park Y.U., Jeong J., Lee H., Young Mun J., Kim J.H., Seo Lee J., Dang Nguyen M., Sik Han S., Suh P.G., Ki Park S. (2010). *Disrupted-in-schizophrenia 1* (DISC1) plays essential roles in mitochondria in collaboration with Mitofilin. Proc. Natl. Acad. Sci. U.S.A..

[59] Cataldo A.M., McPhie D.L., Lange N.T., Punzell S., Elmiligy S., Ye N.Z., Froimowitz M.P., Hassinger L.C., Menesale E.B., Sargent L.W. (2010). Abnormalities in mitochondrial structure in cells from patients with bipolar disorder. Am. J. Pathol..

[60] Ferger A.I., Campanelli L., Reimer V., Muth K.N., Merdian I., Ludolph A.C., Witting A. (2010). Effects of mitochondrial dysfunction on the immunological properties of microglia. J. Neuroinflamm..

[61] Li X., Chauhn A., Shiekh A.M., Patil S., Chauhn V., Li X.M., Ji L., Brown T., Malika M. (2009). Elevated immune response in the brain of autistic patients. J. Neuroimmunol..

[62] Nakamura K., Sekine Y., Ouchi Y., Tsujii M., Yoshikawa E., Futatsubashi M., Tsuchiya K.J., Sugihara G., Iwata Y., Suzuki K. (2010). Brain serotonin and dopamine transporter bindings in adults with high-functioning autism. Am. J. Psychiatry.

[63] Zhong M., Wang X., Xiao J., Yi J., Zhu X., Liao J., Wang W., Yao S. (2011). Amygdala hyperactivation and prefrontal hypoactivation in subjects with cognitive vulnerability to depression. Biol. Psychol..

[64] Berton O., Nestler E.J. (2006). New approaches to antidepressant drug discovery: beyond monoamines. Nat. Rev. Neurosci..

[65] Price J.L., Drevets W.C. (2010). Neurocircuitry of mood disorders. Neuropsychopharmacol. Rev..

[66] Kang H.J., Voleti B., Hajszan T., Rajkowska G., Stockmeier C.A., Licznerski P., Lepack A., Majik M.S., Jeong L.S., Banasr M. (2012). Decreased expression of synapse-related genes and loss of synapses in major depressive disorder. Nat. Med..

[67] Xie W., Cai L., Yu Y., Gao L., Xiao L., He Q., Ren Z., Liu Y. (2014). Activation of brain indoleamine 2,3-dioxygenase contributes to epilepsy-associated depressive-like behavior in rats with chronic temporal lobe epilepsy. J. Neuroinflamm..

[68] Steiner S., Walter M, Gos T., Guillemin G.J., Bernstein H.G., Sarnyai Z., Mawrin C., Brisch R., Bielau H., zu Schwabedissen L.M. (2011). Severe depression is associated with increased microglial quinolinic acid in subregions of the anterior cingulate gyrus: evidence for an immune-modulated glutamatergic neurotransmission?. J. Neuroinflamm..

[69] Fattore L., Melis M., Fadda P., Pistis M., Fratta W. (2010). The endocannabinoid system and nondrug rewarding behaviours. Exp. Neurol..

[70] Müller N., Schwarz M.J., Dehning S., Douhe A., Cerovecki A., Goldstein-Muller B., Spellmann I., Hetzel G., Maino K., Kleindienst N. (2006). The cyclooxygenase-2 inhibitor celecoxib has therapeutic effects in major depression: results of a double-blind, randomized, placebo controlled, add-on pilot study to reboxetine. Mol. Psychiatry.

[71] Abbasi S.H., Hosseini F., Modabbernia A., Ashrafi M., Akhondzadeh S. (2012). Effect of celecoxib add-on treatment on symptoms and serum IL-6 concentrations in patients with major depressive disorder: Randomized double-blind placebo-controlled study. J. Affect. Disord..

[72] Robinson R.G., Price T. (1982). Post-stroke depressive disorders: a follow-up study of 103 Patients. Stroke.

[73] Krishnan K.R.R., Hays J.C., Blazer D.G. (1997). MRI-defined vascular depression. Am. J. Psychiatry.

[74] Fujikawa T., Yamawaki S., Touhouda Y. (1993). Incidence of silent cerebral infarction in patients with major depression. Stroke.

[75] Alexopoulos G.S., Bruce M.L., Silbersweig D., Kalayam B., Stern E. (1999). Vascular depression: a new view of late-onset depression. Dialogues Clin. Neurosci..

[76] Taylor W.D., Aizenstein H.J., Alexopoulos G.S. (2013). The vascular depression hypothesis: mechanisms linking vascular disease with depression. Mol. Psychiatry.

[77] Naismith S.L., Norrie L.M., Mowszowski L., Hickie I.B. (2012). The neurobiology of depression in later-life: Clinical, neuropsychological, neuroimaging and pathophysiological features. Prog. Neurobiol..

[78] Geerlings M.I., Koudstaal P.J., Hofman A., Breteler M.M.B. (2008). History of depression, depressive symptoms, and medial temporal lobe atrophy and the risk of Alzheimer disease. Neurology.

[79] Rosenberg P.B., Lyketsos C.G. (2008). Mild cognitive impairment: searching for the prodrome of Alzheimer’s disease. World Psychiatry.

[80] Zlokovic B.V. (2011). Neurovascular pathways to neurodegeneration in Alzheimer’s disease and other disorders. Nat. Rev. Neurosci..

[81] Jackson S.P. (2011). Arterial thrombosis—Insidious, unpredictable and deadly. Nat. Med..

[82] Phillipson M., Kubes P. (2011). The neutrophil in vascular inflammation. Nat. Med..

[83] Abramsson A., Lindblom P., Betsholtz C. (2003). Endothelial and nonendothelial sources of PDGF-B regulate pericyte recruitment and influence vascular pattern formation in tumors. J. Clin. Invest..

[84] Darland D.C., Massingham L.J., Smith S.R., Piek E., Saint-Geniez M., D’Amorea P.A. (2003). Pericyte production of cell-associated VEGF is differentiationdependent and is associated with endothelial survival. Dev. Biol..

[85] Argaw A.T., Asp L., Zhang J., Navrazhina K., Pham T., Mariani J.N., Mahase S., Dutta D.J., Seto J., Kramer E.G. (2012). Astrocyte-derived VEGF-A drives blood-brain barrier disruption in CNS inflammatory disease. J. Clin. Invest..

[86] Hamdan R., Zhou Z., Kleinerman E.S. (2011). SDF-1a Induces PDGF-B Expression and the differentiation of bone marrow cells into pericytes. Mol. Cancer Res..

[87] Ödemis V., Boosmann K., Heinen A., Küry P., Engele1 J. (2010). CXCR7 is an active component of SDF-1 signalling in astrocytes and Schwann cells. J. Cell Sci..

[88] Song N., Huang Y., Shi H., Yuan S., Ding Y., Song X., Fu Y., Luo Y. (2009). Overexpression of platelet-derived growth factor-BB increases tumor pericyte content via stromal-derived factor-1A/CXCR4 axis. Cancer Res..

[89] Bonkowski D., Katyshev V., Balabanov R.D., Borisov A., Dore-Duffy P. (2011). The CNS microvascular pericyte: pericyte-astrocyte crosstalk in the regulation of tissue survival. Fluids Barriers CNS.

[90] Abbott N.J., Ronnback L., Hansson E. (2006). Astrocyte–endothelial interactions at the blood–brain barrier. Nat. Res. Neurosci..

[91] Lavretsky H., Meeks T. (2009). Late-life depression: managing mood in patients with vascular disease. Cur. Psychiatry.

[92] Namekawa Y., Baba H., Maeshima H., Nakano Y., Satomura E., Takebayashi N., Nomoto H., Suzuki T., Arai H. (2013). Heterogeneity of elderly depression: Increased risk of Alzheimer’s disease and Aβ protein metabolism. Prog. Neuro-Psychopharmacol. Biol. Psychiatry.

[93] Levy R.L., Langer S.L., Whitehead W.E. (2007). Social learning contributions to the etiology and treatment of functional abdominal pain and inflammatory bowel disease in children and adults. World J. Gastroenterol..

[94] Wiech K., Ploner M., Tracey I. (2008). Neurocognitive aspects of pain perception. Trends Cogn. Sci..

[95] Uceyler N., Rogausch J.P., Toyka K.V., Sommer C. (2007). Differential expression of cytokines in painful and painless neuropathies. Neurology.

[96] Backonja M.M., Coe C.L., Muller D.A., Schell K. (2008). Altered cytokine levels in the blood and cerebrospinal fluid of chronic pain patients. J. Neuroimmunol..

[97] Tracey I. (2010). Getting the pain you expect: mechanisms of placebo, nocebo and reappraisal effects in humans. Nat. Med..

[98] Apkarian A.V., Sosa Y., Sonty S., Levy R.M., Harden R.N., Parrish T.B., Gitelman D.R. (2004). Chronic back pain is associated with decreased prefrontal and thalamic gray matter density. J. Neurosci..

[99] Zubieta J.K., Bueller J.A., Jackson L.R., Scott D.J., Xu Y., Koeppe R.A., Nichols T.E., Stohler C.S. (2005). Placebo effects mediated by endogenous opioid activity on mu-opioid receptors. J. Neurosci..

[100] Lorenz J., Minoshima S., Casey K.L. (2003). Keeping pain out of mind: The role of the dorsolateral prefrontal cortex in pain modulation. Brain.

[101] Maizels M., Aurora S., Heinricher M. (2012). Beyond neurovascular: migraine as a dysfunctional neurolimbic pain network. Headache.

[102] Giuffrida A., Leweke F.M., Gerth C.W., Schreiber D., Koethe D., Faulhaber J., Klosterkotter J., Piomelli D. (2004). Cerebrospinal anandamide levels are elevated in acute schizophrenia and are inversely correlated with psychotic symptoms. Neuropsychopharmacology.

[103] Leweke F.M., Piomelli D., Pahlisch F., Muhl D., Gerth C.W., Hoyer C., Klosterkotter J., Hellmich M., Koethe D. (2012). Cannabidiol enhances anandamide signaling and alleviates psychotic symptoms of schizophrenia. Transl. Psychiatry.

[104] Blaas K. (2008). Treating depression with cannabinoids. Cannabinoids.

[105] Freund T.F., Katona I., Piomelli D. (2003). Role of endogenous cannabinoids in synaptic signaling. Physiol. Rev..

[106] Varga K., Wagner J.A., Bridgen T., Kunosi G. (1998). Platelet- and macrophage-derived endogenous cannabinoids are involved in endotoxin-induced hypotension. FASEB J..

[107] Carlisle S.J., Marciano-Cabral F., Staab A., Ludwick C., Cabral G.A. (2002). Differential expression of the CB_2_ cannabinoid receptor by rodent macrophages and macrophage-like cells in relation to cell activation. Int. Immunopharmacol..

[108] Han K.H., Lim S., Ryu J., Lee C.W., Kim Y., Kang J.H., Kang S.S., Ahn Y.K., Park C.S., Kim J.J. (2009). CB_1_ and CB_2_ cannabinoid receptors differentially regulate the production of reactive oxygen species by macrophages. Cardiovasc. Res..

[109] Velasco G., Galve-Roperh I., Sanchez C., Blazquez C., Haro A., Guzman M. (2005). Cannabinoids and ceramide: two lipids acting hand-by-hand. Life Sci..

[110] Atwood B.K., Wager-Miller J., Haskins C., Straiker A., Mackie K. (2012). Functional selectivity in CB_2_ cannabinoid receptor signaling and regulation: Implications for the therapeutic potential of CB_2_ ligands. Mol. Pharmacol..

[111] Klein T.W., Newton C.A., Nakachi N., Friedman H. (2000). Δ^9^-Tetrahydrocannabinol treatment suppresses immunity and early IFN-gamma, IL-12, and IL-12 receptor β2 responses to *Legionella pneumophila* infection. J. Immunol..

[112] Newton C.A, Chou P.J., Perkins I., Klein T.W. (2009). CB_1_ and CB_2_ cannabinoid receptors mediate different aspects of Δ^9^-tetrahydrocannabinol (THC)-induced T helper cell shift following immune activation by *Legionella Pneumophila* infection. J. Neuroimmune. Pharmacol..

[113] Paintlia A.S., Paintlia M.K., Singh I., Singh A.K. (2006). IL-4-induced peroxisome proliferator-activated receptor gamma activation inhibits NF-κB *Trans* activation in central nervous system (CNS) glial cells and protects oligodendrocytes progenitors under neuroinflammatory disease conditions: implication for CNS-demyelinating diseases. J. Immunol..

[114] Galve-Roperh I., Sanchez C., Cortes M.L., Del Pulgar T.G., Izquierdo M., Guzman M. (2000). Anti-tumoral action of cannabinoids: involvement of sustained ceramide accumulation and extracellular signal-regulated kinase activation. Nat. Med..

[115] Holland W.L., Miller R.A., Wang Z.V., Sun K., Barth B.M., Bui H.H., Davis K.E., Bikman B.T., Halberg N., Rutkowski J.M. (2011). Receptor-mediated activation of ceramidase activity initiates the pleiotropic actions of adiponectin. Nat. Med..

[116] Bensinger S.J., Tontonoz P. (2008). Integration of metabolism and inflammation by lipid-activated nuclear receptors. Nature.

[117] Rutherford C., Childs S., Ohotski J., McGlynn L., Riddick M., MacFarlane S., Tasker D., Pyne S., Pyne N.J., Edwards J. (2013). Regulation of cell survival by sphingosine-1-phosphate receptor S1P1 via reciprocal ERK-dependent suppression of Bim and PI-3-kinase/protein kinase C-mediated upregulation of Mcl-1. Cell Death Dis..

[118] Wang W., Huang M.C., Goetzl E.J. (2007). Type 1 sphingosine 1-phosphate G protein-coupled receptor (S1P1) mediation of enhanced IL-4 generation by CD4 T cells from S1P1 transgenic mice. J. Immunol..

[119] Choi I.Y., Ju C., Jalin A.M.A.A., Lee D.A., Prather P.L., Kim W.K. (2013). Activation of cannabinoid CB_2_ receptor-mediated AMPK/CREB pathway reduces cerebral ischemic injury. Am. J. Pathol..

[120] Lim C.T., Kola B., Korbonits M. (2010). AMPK as a mediator of hormonal signaling. J. Mol. Endocrinol..

[121] Zhao M., Sun L., Yu X.J., Miao Y., Liu J.J., Wang H., Ren J., Zang W.J. (2013). Acetylcholine mediates AMPK-dependent autophagic cytoprotection in H9c2 cells during hypoxia/reoxygenation injury. Cell. Physiol. Biochem..

[122] Rockwell C.E., Snider N.T., Thompson J.T., Vanden Heuvel J.P., Kaminski N.E. (2006). Interleukin-2 suppression by 2-arachidonyl glycerol is mediated through peroxisome proliferator-activated receptor- gamma independently of cannabinoid receptors 1 and 2. Mol. Pharmacol..

[123] Andersson U., Filipsson K., Abbott C.R., Woods A., Smith K., Bloom S.R., Carling D., Small C.J. (2004). AMP-activated protein kinase plays a role in the control of food intake. J. Biol. Chem..

[124] Huypens P., Moens K., Heimberg H., Ling Z., Pipeleers D., Van de Casteele M. (2005). Adiponectin-mediated stimulation of AMP-activated protein kinase (AMPK) in pancreatic β cells. Life Sci..

[125] Recchiuti A., Serhan C.N. (2012). Pro-resolving lipid mediators (SPMs) and their actions in regulating miRNA in novel resolution circuits in inflammation. Front. Immunol..

[126] Krishnamoorthy S., Recchiuti A., Chiang N., Fredman G., Serhan C.N. (2012). Resolvin D1 receptor stereoselectivity and regulation of inflammation and proresolving microRNAs. Am. J. Pathol..

[127] Titos E., Rius B., Gonzalez-Periz A., Lopez-Vicario C., Moran-Salvador E., Martinez-Clemente M., Arroyo V., Claria J. (2011). Resolvin D1 and its precursor docosahexaenoic acid promote resolution od adipose tissue inflammation by eliciting macrophage polarization toward an M2-like phenotype. J. Immunol..

[128] Le Y., Gong W., Tiffany H.L., Tumanov A., Nedospasov S., Shen W., Dunlop N.M., Gao J.L., Murphy P.M., Oppenheim J.J. (2001). Amyloid β42 activates a G-protein-coupled chemoattractant receptor, FPR-Like-1. J. Neurosci..

[129] Amminger G.P., Schäfer D.M.R., Papageorgiou K., Klier C.M., Cotton S.M., Harrigan S.M., Mackinnon A., McGorry P.D., Berger G.E. (2010). Long-chain ω-3 fatty acids for indicated prevention of psychotic disorders. Arch. Gen. Psychiatry.

[130] Emsley R., Myburgh C., Oosthuizen P., van Rensburg S.J. (2002). Randomized, placebo-controlled study of ethyl-eicosapentaenoic acid as supplemental treatment in schizophrenia. Am. J. Psychiatry.

[131] Baruch K., Ron-Harel N., Gal H., Deczkowska A., Shifrut E., Ndifon W., Mirlas-Neisberg N., Cardon M., Vaknin I., Cahalon L. (2013). CNS-specific immunity at the choroid plexus shifts toward destructive Th2 inflammation in brain aging. Proc. Natl. Acad. Sci. USA..

[132] Fenn A.M., Hall J.C.E., Gensel J.C., Popovich P.G., Godbout J.P. (2014). IL-4 signaling drives a unique arginase^+^/IL-1β^+^ microglia phenotype and recruits macrophages to the inflammatory CNS: Consequences of age-related deficits in IL-4Rα after traumatic spinal cord injury. J. Neurosci..

